# Using a non-surgical transcutaneous intraglandular injection technique to deliver cell and cell-free therapies to murine submandibular salivary glands

**DOI:** 10.1371/journal.pone.0326769

**Published:** 2025-07-10

**Authors:** Arvind Hariharan, Janaki Iyer, Akram Almansoori, Younan Liu, Meet Shah, Piotr Pater, Tyler Lalonde, Simon D. Tran

**Affiliations:** 1 Faculty of Dental Medicine and Oral Health Sciences, McGill University, Montréal, Québec, Canada; 2 Medical Physics Unit, Bronfman Department of Oncology, McGill University, Montréal, Québec, Canada; 3 Small Animal Imaging Labs Services (SAIL) Platform, Research Institute-McGill University Health Center (RI-MUHC), Montréal, Québec, Canada; National Institutes of Health, UNITED STATES OF AMERICA

## Abstract

Salivary Glands (SGs) are vital organs that are particularly prone to damage due to head and neck cancer radiation treatment, as well as other autoimmune disorders. SG hypofunction often impairs the production of saliva, which causes a significant loss of functioning for the oral cavity. There are a multitude of treatments for rescuing SG hypofunction; however, they are usually administered systemically, causing adverse effects and requiring higher doses of the agents. More localised approaches have demonstrated adequate potential as alternative treatments but are still in their initial stages of investigation. This study proposes a localised delivery technique termed as the ‘Intraglandular (IG) Non-Surgical Transcutaneous Injection,’ to deliver cell and cell-free therapies into the submandibular salivary glands (SMGs) of mice. First, we used anatomical landmarks, radiography, and tissue dissection to devise a method to localise the center of the SMGs with either a pen marker or a customized plastic stent. Second, we used a dye (trypan blue) to verify the accuracy of the injection technique to the SMGs. Third, we injected Green Fluorescent Protein Bone Marrow Mesenchymal Stem Cells (GFP + ve BM-MSC) and tracked them for 7 days in SMGs to further demonstrate that the cells were embedded into SMG tissue and that our injection technique did not injure the glandular morphology. We therefore hypothesize that this IG technique would provide a less invasive, lower dose and highly accurate administration of cell and cell-free therapies to hypofunctional mice SMGs.

## Introduction

Salivary Glands (SGs) are vital for the oral cavity because they produce saliva that assist with important functions like lubrication, speech, mastication, and protection of the oral structures. Unfortunately, SGs are prone to damage, particularly due to irradiation (IR) and autoimmune disorders such as Sjogren’s Syndrome (SS), which impairs the production of saliva and in turn, reduces oral health [1]. To treat SG damage, the most common route of administering agents is through systemic routes such as intravenous or subcutaneous injections. Despite technological advancements in systemic administration, they still lack reliability due to reduced activity at the site owing to systemic absorption and associated adverse effects [2,3]. Moreover, systemic routes require a higher dose for absorption into the site of action. For example, studies have shown that systemic treatment with Mesenchymal Stem Cells (MSCs) result in approximately 99% of the cells being absorbed into the lungs [4]. Recently, more localised approaches, such as the intraductal (retroductal), oral, topical and intraglandular (IG) routes are being investigated for the treatment of SG disorders. These routes have demonstrated great potential in reducing adverse effects and contributing to a more rapid accumulation of drugs and cell therapies in the SGs.

The retroductal route has been used for agents such as radioprotective drugs like Amifostine (AMI) and other cell-based therapies. A recent study by Varghese *et al.* demonstrated that retroductally administered AMI successfully restored salivary flow and acinar cell regeneration while reducing hypotension in mice, in comparison to the intravenous route [5]. More recently, Kasamatsu *et al.* evaluated this method of delivery in atrophic SGs of irradiated rats using cultured rat SG cells and found that salivary secretion was restored [6]. The retroductal route has also been effectively tested in patients in both the parotid and submandibular glands (SMGs) with sialography imaging techniques and to treat inflammatory conditions [3,7]. While successful, the retroductal technique requires expertise and specialized equipment to cannulate the Wharton’s duct, which makes it technically demanding especially in smaller animals like mice [8]. Also, there is potential for the retroductal technique to be invasive in mice as it can cause local irritation, duct obstruction and swelling [8].

The oral and topical routes have been less commonly used because of the tendency of agents to lose their bioactivity [9]. Recent studies have employed the use of nanoparticles or hydrogels for the controlled release of drugs by these routes with reasonable success [9,10]. Studies using mucoadhesive polymers and hydrogels incorporating Pilocarpine have been tested on patients with SS as buccal inserts and have shown that salivary secretions were increased with adequate patient tolerance [11]. However, the usage of these encapsulating membranes are still in their preliminary stages due to reports of initial burst of agents and modifications in their mechanical properties [9,12].

The IG approach for the treatment of SG disorders is gaining importance rapidly. The primary advantage of this technique is that it provides direct access to the SGs in comparison to the other modes of administration mentioned earlier [3]. Using this route is also less time-consuming and the dose of agents required is less compared to systemic administration [3]. The IG approach is not a new technique and is commonly used for the treatment of sialorrhea with Botulinum Toxin [13]. It is also under investigation in clinical trials for the use of SCs in the treatment of SG damage [14]. The two main types of IG approaches are the surgical and non-surgical routes.

The first instance of the IG technique involved using a surgical approach. Typically, it involves a conservative, horizontal incision to expose the SGs of interest [3,15]. Initial studies using the surgical IG technique involved the use of botulinum toxin for the treatment of Sialorrhea [16]. The technique was later used for bone marrow SC transplantation and their extracts into the SGs [15]. However, while successful, there have been some complications associated with the surgical approach. Bushara *et al.* found that it could be associated with carotid artery and facial nerve injury when used for the parotid glands [16]. Also, the tolerance levels, especially in the pediatric population, and the high costs have reduced the technique’s popularity [17].

Currently, the preferred method of IG injections is the non-surgical technique. It is a well-established technique, particularly for the treatment of sialorrhea, and has evolved with the use of ultrasound-guided (USG) transcutaneous injections [13,18]. Earlier non-surgical techniques relied on anatomical landmark identification; however, studies have shown that the introduction of USG techniques improved accuracy [19]. Clinical trials are currently underway using the USG IG injection to deliver various types of MSCs into irradiated SGs [14,20]. The initial results from the MESRIX trial involving Adipose tissue MSCs have shown restoration of salivary function for patients undergoing radiotherapy with fewer side effects [20].

While the USG technique is well-established, the use of the ultrasound is heavily operator-dependent and is associated with increased costs of treatment. Also, the availability of ultrasound transducer probes for usage in pre-clinical animal studies is sparse, warranting a more feasible IG injection technique. To address these issues and to minimize trauma associated with the surgical IG technique, we developed a non-surgical transcutaneous IG technique for the SMGs. This technique was developed with the aid of both anatomical and radiological landmarks as well as tissue dissection. The accuracy of the IG technique was further validated across different mouse strains using Trypan Blue Dye. Finally, using green fluorescent protein (GFP + ve) labelled mouse bone marrow MSCs (BM-MSCs), we were able to demonstrate that these cells could be detected within the tissue for seven days, indicating their absorption into the SG tissue with the non-surgical transcutaneous IG injection.

## Materials and methods

The protocol described in this peer-reviewed article is published on protocols.io and is included for printing as S1 File with this article. The step-by-step protocol is in the following link: https://dx.doi.org/10.17504/protocols.io.81wgbkjj3gpk/v1.

### Mice

C57BL/6 and NOD mice (female), 20 weeks of age, were obtained from the Comparative Medicine and Animal Resources Center (CMARC) animal facilities, derived from Charles River and Taconic Laboratories. Mice were housed in clean conditions with ad libitum access to food and autoclaved distilled water supplied by the CMARC.

### Ethics statement

This protocol was conducted on mice, following all the guidelines laid by the Canadian Council on Animal Care and under protocols approved by the McGill University Animal Care Committee (Protocol #5330). Animal experiments conformed to the ARRIVE 2.0 guidelines. The procedure was performed under Isoflurane inhalation anaesthesia and all efforts were made to minimize suffering.

### Intraglandular non-surgical transcutaneous injection procedure

**Fig 1:**
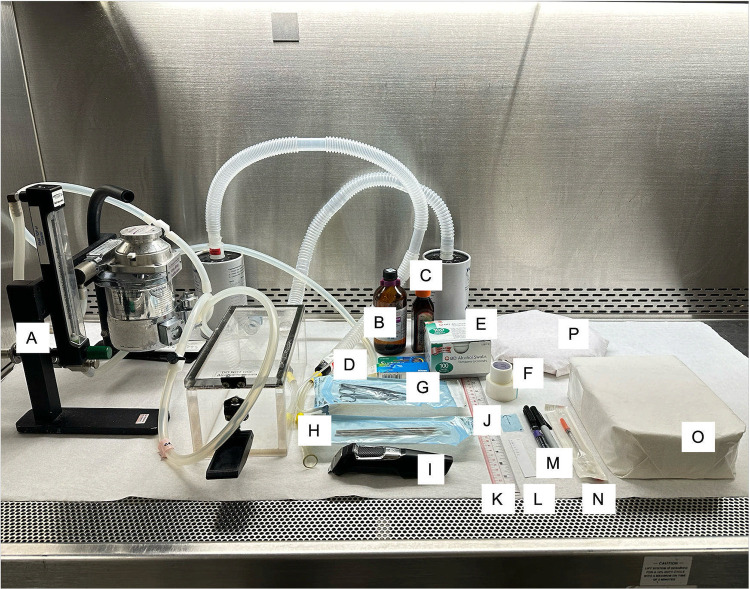
Armamentarium for transcutaneous IG injection procedure. In a clean, disinfected hood, the following armamentarium were prepared: A. Inhalation anaesthetic unit; B. Isoflurane-USP; C. 10% Povidine-iodine solution; D. Lubricant ophthalmic ointment; E. 70% Ethanol swabs; F. Masking tapes; G. Surgical scissors, stainless steel; H. Anatomical forceps, fine, stainless steel; I. Electric razor; J. Disinfected plastic stencil; K. Disinfected ruler; L. Paper ruler; M. Fine tip markers; N. 28G, 12.7 mm, 0.5 ml, U-100 insulin syringe; O. Working platform; P. Heated recovery pad.

The non-surgical transcutaneous IG injection procedure was performed under aseptic conditions. All the surgical material was sterilized by autoclave, and all the surfaces were disinfected with a 1:10 dilution of commercial household bleach and 70% ethanol. The operators donned personal protective equipment that included disposable laboratory coats, goggles, gloves, head caps, and masks. The required instruments and materials (Fig 1) for the procedure were organized within the sterile field of the hood.

**A. Sedation:**

**Fig 2:**
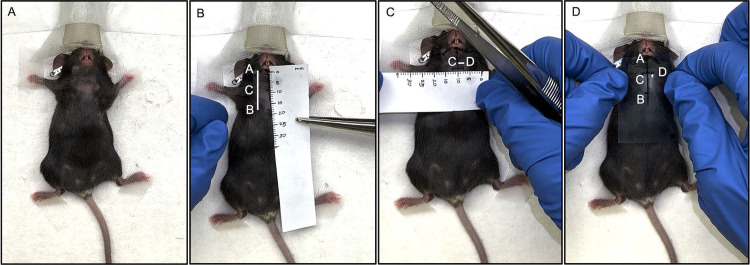
Anatomical Landmark Identification. A. The cervical area of a sedated and restrained mouse was shaved and prepared for the procedure; B. Mark the midline from the lower lip (point A) to point B at the xiphoid process of the sternum (length of 19 mm) was traced, point C was marked 9 mm inferiorly to point A; C. The sites of IG injections (points D) were measured 2 mm laterally on each side at point C; D. A plastic stencil was created as a guide for injections, using the above landmarks and measurements.

C57BL/6 (female) mice of age 20 weeks, weight ranging from 15–20 grams were sedated using the following procedure:

- The mouse was deeply sedated with an inductive dose of 4% (vol/vol) Isoflurane using an Isoflurane induction chamber (VetEquip Inc., USA). The sedated mouse was then transferred onto an aseptic working platform in supine position, and a
  maintenance dose of 2% (vol/vol) Isoflurane was administered using a nose cone. The depth of anesthesia was evaluated by the toe pinch reflex technique. To avoid corneal drying, ophthalmic ointment was used. The mouse was restrained into position with masking tapes, at a comfortable level of the nose cone exposing the lower lip and chin region. Then the cervical area of the mouse (from the lower lip to approximately the sternum of the chest) was swabbed with 70% Ethanol, shaved, and cleaned with Povidine-iodine (Fig 2A and S1 Video).

**B. Identification of anatomical landmarks to localize the submandibular glands:**

The following anatomical landmarks were marked on the mouse, using a fine-tipped marker (Fig 2B, C, D and S2 Video):

a. A line was traced from the midline of the lower lip (point A), passing through the inferior border of the mandible to the xiphoid process of the sternum (point B). The total length of the line joining points A to B is 19 mm. This was now called ‘Line AB.’ (Fig 2B).
b. The lower border of the mandible was palpated and traced from Point A to the angle of the mandible on either side. The superior border of the SMGs was measured to be at 4 mm inferior to the angle of the mandible, bilaterally.
c. From point A, another point (point C) was traced, 9 mm inferiorly (towards the sternum) along the midline (Fig 2B). Point C is the point on Line AB, from where the center of the SMG could be traced laterally on both sides. The distance between Point A and Point C was now, ‘Line AC.’
d. The site of IG injections was measured 2 mm laterally on each side at Point C. These two points coincided with the center of the SMGs bilaterally (Point D) (Fig 2C). The distance between Point C and D was now, ‘Line CD,’
e. For ease of reproducibility of these landmarks, our team developed a plastic stencil with the aforementioned measurements. This stencil was prepared using an autoclavable plastic sheet, wherein the landmarks and measurements were duplicated and used as a guide for the injection (Fig 2D).
**C. Injection technique:**

**Fig 3:**
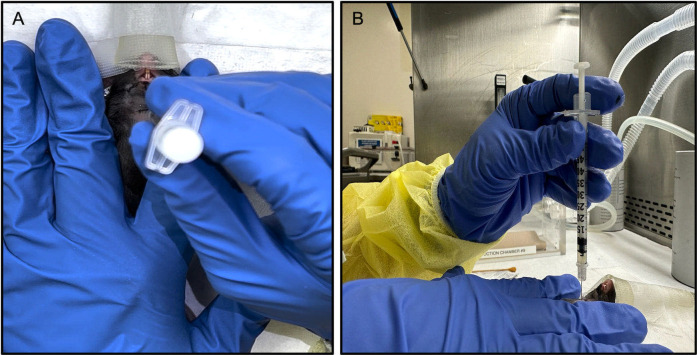
Intraglandular Injection Technique. The syringe loaded with the solution was placed at one IG injection site, perpendicular to the mouse (A. Bird’s eye view; B. Lateral view).

The loaded syringe was placed at one IG injection site (Point D), perpendicular to the mouse. The needle was inserted to a depth of 3 mm to reach the center of the SMG (Fig 3A, B and S3 Video). The solution was gradually deposited during the withdrawal of the syringe. Then the area of injection was gently massaged to dissipate the solution. (Note that the needle was allowed to proceed past the recommended depth of 3 mm and then gently withdrawn back upwards before depositing the suspension, to avoid gland perforation). This step of non-surgical transcutaneous IG injection was repeated on the other side, after which the Isoflurane was stopped, and the mouse was transferred to a recovery hot pad. The mouse was initially monitored every hour for 5 hours and then daily. This same procedure was repeated on the other mice.

## Results and discussion

To validate the reproducibility of the identified landmarks and measurements across species, the location of the SMGs was verified in both C57Bl/6 and non-obese diabetic (NOD) female mice (n = 5 each). NOD mice were also used because they are commonly employed as the mouse model to study SG hypofunction in SS. It was found that there was a 1 mm difference in the measurement of the midline (Point A to Chest Wall (Table 1). As a result, the distance between Point A and Point B also had a difference of 1 mm. However, it was noted that the skin thickness and SG thickness were the same across both mouse species.

**Table 1:** Measurements as plotted on C57BL/6 and NOD mice.

Landmarks measured	Distance/thickness (C57BL/6) (n = 5)	Distance/thickness (NOD) (n = 5)
Mandible to Chest wall	17 mm	15 mm
Line AB (Lower lip to Xiphoid Process)	19 mm	18 mm
Mandible to SMG center	7 mm	6 mm
Line AC (Lower lip to SMG center)	9 mm	8 mm
Line CD (Midline to SMG center)	2 mm	2 mm
Skin and fascia thickness	1 mm	1 mm
SMG thickness	3 mm	3 mm

Following landmark identification, the location of the SMGs was verified by injecting 20 ul of 0.4% Trypan Blue Stain (Gibco, USA) at the center of the gland at 90^o^ angle, following the injection protocol mentioned [3]. The SMGs were then harvested and on inspection, it was found that the Trypan Blue entered the gland with a success rate of 80%, as reported previously [3].

**Fig 4:**
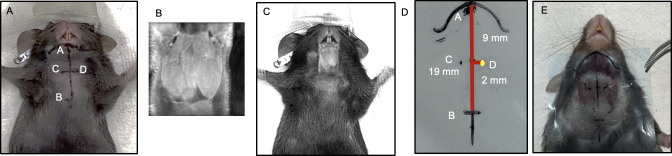
Further Validation of the Location of Submandibular Glands using an MRI Image and Plastic Stencil. A. Sedated and restrained mouse with plotted landmarks (Points A, B, C, and D) for the non-surgical transcutaneous IG injections; B. T2-weighted MRI image of the mouse SMG. C. superimposed image of our landmarks and the frontal cross-sectional T2-weighted MRI image of the mouse SMG; D. A plastic stencil prepared with the same landmarks. E. Image of the exposed SMGs with the marked stencil placed.

To further verify the accuracy of our defined anatomical landmarks and improve the success rate, we obtained a superimposed image of our landmarks and a frontal cross-sectional T2-weighted MRI image of the mouse SMG area (Fig 4). Mice were anesthetized with isoflurane (2–3% for induction, 1–2% for maintenance) and placed on a heated MRI bed to maintain body temperature at 37°C. Respiratory and heart rate were monitored throughout the scan using a mouse-specific physiological monitoring system. Imaging was performed using a Bruker 7T MRI system (BioSpec 70/30) with a volume mouse head coil. A 3D T2-weighted imaging sequence based on SSFP (Steady-State Free Precession) was employed. The field of view was set to 15 x 15 x 6 mm, and the resolution was 0.091 x 0.091 x 0.073 mm. Imaging parameters included an echo time (TE) of 3.298 ms, a repetition time (TR) of 6.596 ms, and 4 repetitions with 2 averages. The scan was performed without fat suppression to visualize the salivary glands. The protocol was designed for high-resolution imaging with good contrast between tissues. The total scan time was approximately 45 minutes.

**Fig 5:**
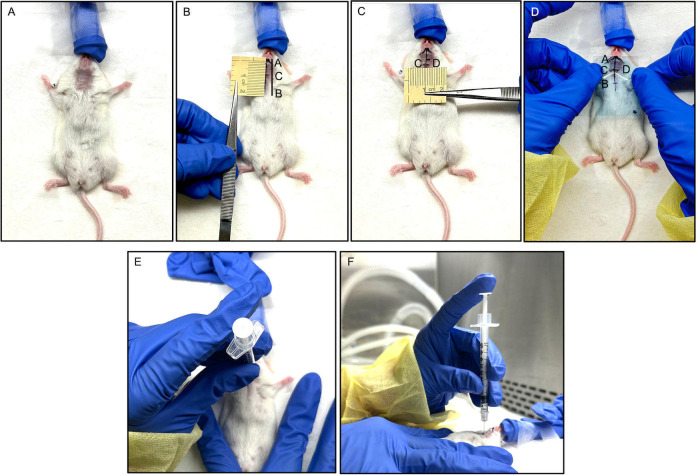
Submandibular Salivary Gland Localization and Injection in NOD Mice. A. The cervical area of a sedated and restrained mouse was shaved and prepared for the procedure; B. Mark the midline from the lower lip (point A) to point B xiphoid process of the sternum (length of 18 mm) and point C was traced, 8 mm inferiorly to point A; C. The sites of IG injections (points D) were measured 2 mm laterally on each side at point B; D A plastic stencil was created as a guide for injections, using the above landmarks and measurements; E, F. The syringe loaded with the solution was placed at one IG injection site, perpendicular to the mouse (E. Bird’s eye view; F. Lateral view).

**Fig 6:**
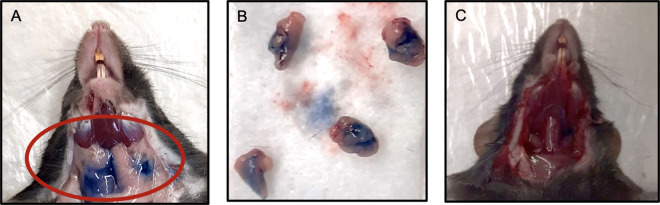
Verification of salivary gland location using Trypan Blue Dye. A. Exposed SMGs of a euthanized mouse after non-surgical, transcutaneous IG Trypan Blue injections; B. Harvested SMGs revealed uptake of the Trypan Blue dye; C. SMG area after excision of the glands, showing no spread of dye to the surrounding structures.

Once the landmarks and their measurements were confirmed, a plastic stencil with the plotted landmarks was prepared (Fig 4D). We believed that the preparation of this stencil would aid us in saving time to plot landmarks and inject as well as improve the accuracy. The Trypan Blue dye injections were repeated with both C57Bl/6 and NOD mice (n = 10 each) using the stencil, and we found that the success rate of dye uptake improved to 100%, with minimal spread of the dye outside the glands (Figs 5 and 6).

**Fig 7:**
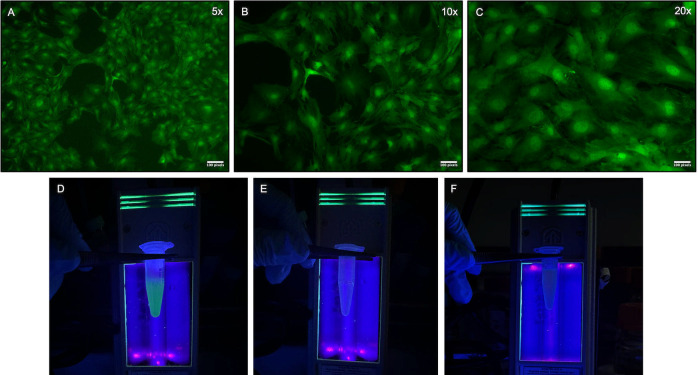
Intraglandular injection of GFP + bone marrow mesenchymal stem cells in submandibular glands. A-C; Confocal microscopy images demonstrating GFP + ve BM-MSCs in culture (5x, 10x, 20x; scale bar = 100 pixel = 26400.58 micron). D; Eppendorf tube with GFP + ve BM-MSCs emitting green fluorescence under ultra-violet lamp. E; Control Eppendorf tube with normal saline emitting no colour under ultra-violet lamp. F; Control Eppendorf tube with non-labelled MSCs emitting no colour under ultra-violet lamp.

Following the stencil preparation, the pilot study using the GFP + ve BM-MSCs was performed. The GFP + ve BM-MSCs utilized in this study were derived from GFP+ mice (C57BL/6-Tg(ACTB-EGFP)l0sb/J, Jackson Laboratory, USA). Following protocols, [15,21] the GFP + ve BM-MSCs were cultured and expanded in 100-mm dishes with alpha-MEM (12571048, Gibco, Fisher Scientific) supplemented with 10% fetal bovine serum and 1% penicillin/streptomycin (10,000 U/ml). The media was changed every 2–3 days. The cultured GFP + ve BM-MSCs were monitored daily and imaged using confocal microscopy, to determine structural and morphological changes (Fig 7). Upon attaining 75% confluence, these cells were detached with 0.25% Trypsin (25200056, GibcoThermoFisher Scientific) and centrifuged at 1,200 rpm for 5 minutes at 4^o^C. They were then re-suspended in 0.9% normal saline, to prepare for the transcutaneous IG injections. Each syringe was loaded at a concentration of 2x10^6^ cells in 50ul saline.

The GFP + BM-MSCs were injected using the above IG protocol with the stencil into the SMGs of 6 mice and were followed up for a week. No adverse reactions were observed at this time. On Days 1, 3, and 7 post-injection, mice (n = 2 each) were sacrificed, and the SMGs were harvested and fixed in 4% Paraformaldehyde (P6148, Sigma-Aldrich) and embedded in paraffin. The serial sections were sliced into 4µm thickness. These sections were studied as both unstained and Hematoxylin and Eosin-stained sections (H&E). Conversely, the unstained serial sections when viewed under the confocal microscope (ZeissLeica SP8 (Leica, Germany). To evaluate the pattern of distribution of the locally injected GFP + ve cells, images were acquired using the objective Plan-Apochromat 20x, with Alexa Fluor 888 for signal detection. The surrounding salivary epithelial cells were stained with Pan Cytokeratin (Pan CK) marker (MA5–13203, Thermo Fisher Scientific, USA) with Alexa-Fluor 594-conjugated Affinipure^TM^ Fab Fragment Donkey Anti-Mouse IgG (Jackson ImmunoResearch Laboratories Inc, USA).

**Fig 8:**
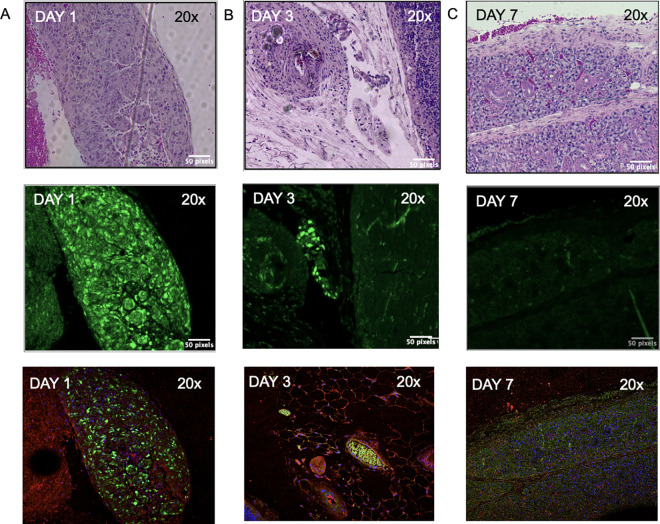
Histopathological Analysis of injected GFP + BM-MSCs in submandibular glands. H&E sections demonstrate normal glandular architecture, whereas the unstained sections show green fluorescence due to GFP + ve MSC injections. Specimens were harvested on Day 1(A), Day 3 (B), and Day 7 (C) post IG injection. The green fluorescence is noticeably fading over time. The third row of images show the same sections with immunofluorescent staining of salivary epithelial cells (Pan CK, red; DAPI, blue) with the GFP+ MSC’s present.

On viewing the histopathological slides under the confocal microscope (Fig 8), the BM-MSCs cells were easily visible (strong GFP green signal) on Day 1 and Day 3, and less visible on Day 7 (fainter green signal). They could be seen interspersed with salivary epithelial cells (Pan CK red and DAPI blue signal) on Day 1, Day 3 and Day 7.

There are some limitations that are anticipated. Further studies of the systemic organs such as the lung, liver, kidney, and spleen are required to confirm the systemic uptake of GFP + ve cells. Another limitation could be the injection of substances with higher viscosity (e.g., hydrogels containing therapeutic agents) because it would require a larger needle bore size, which could perforate the SMGs. While USG IG injections will remain the more accurate gold standard option for the SGs, it is not feasible to be used in mice due to the limited availability of equipment and operator-dependency. Therefore, a cost-effective and easily available method of incorporating this technology would benefit the IG non-surgical technique.

## Conclusion

This paper introduces a non-surgical transcutaneous IG injection technique to deliver cell and cell-free therapies into mice SMGs. The administration of cell and cell-free therapies usually involves systemic administration, which can lead to adverse effects and low site-specific absorption. This injection technique allows for a less invasive administration of agents with a reduced dose. Our results using the GFP + ve BM-MSCs and the pilot studies with Trypan Blue Stain demonstrated that different agents could be delivered into the gland with high accuracy and without disturbing the morphology of the gland. Using the stencil as a guide also allowed for reproducible and faster injections. Although the technique does come with its limitations and could benefit with the introduction of easily available ultrasound-guided technology, it does demonstrate a competent, simplified approach to delivering agents with high accuracy and safety.

## Supporting information

S1 Video Mouse Sedation and Preparation for IG Injection. This video shows how to anesthetize the mice, apply the ophthalmic ointment and restrain them in position. It also shows the preparation of the cervical area for landmark identification.
(MP4)

S2 Video Anatomical Landmark Identification. This video shows how to identify and mark the anatomical landmarks for SMG localization.
(MP4)

S3 Video IG injection technique. This video shows the IG injection technique into the right and left SMG.
(MP4)

S1 File Step-by-step protocol, also available on protocols.io. (PDF)
