# Supplementary material for: Using a non-surgical transcutaneous intraglandular injection technique to deliver cell and cell-free therapies to murine submandibular salivary glands
Source: PLoS One. 2025 Jul 10;20(7):e0326769. doi: 10.1371/journal.pone.0326769 (PMC12244703; doi:10.1371/journal.pone.0326769)
Supplement: S1 File — (PDF) [file pone.0326769.s004.pdf]

Jun 23, 2025

Using a Non-Surgical Transcutaneous Intraglandular Injection Technique to Deliver Cell and Cell-Free Therapies to Murine Submandibular Salivary Glands

DOI
dx.doi.org/10.17504/protocols.io.81wgbkjj3gpk/v1

Arvind Hariharan[1], Janaki Iyer[1], Akram Almansoori[1], Younan Liu[1], Meet Shah[1], Piotr Pater[2], Tyler Lalonde[3], Simon D. Tran[1]

[1]Faculty of Dental Medicine and Oral Health Sciences, McGill University, Montréal, QC, Canada;
[2]Medical Physics Unit, Bronfman Department of Oncology, McGill University, Montréal, QC, Canada;
[3]Manager, Small Animal Imaging Labs Services (SAIL) Platform, Research Institute-McGill University Health Center (RI-MUHC), Montréal, QC, Canada

Arvind Hariharan: Co-first author;
Janaki Iyer: Co-first author;

Dr. Simon Tran Lab

Arvind Hariharan
McGill University

OPEN ACCESS

DOI: dx.doi.org/10.17504/protocols.io.81wgbkjj3gpk/v1

Protocol Citation: Arvind Hariharan, Janaki Iyer, Akram Almansoori, Younan Liu, Meet Shah, Piotr Pater, Tyler Lalonde, Simon D. Tran 2025. Using a Non-Surgical Transcutaneous Intraglandular Injection Technique to Deliver Cell and Cell-Free Therapies to Murine Submandibular Salivary Glands. **protocols.io** https://dx.doi.org/10.17504/protocols.io.81wgbkjj3gpk/v1

License: This is an open access protocol distributed under the terms of the **Creative Commons Attribution License**, which permits unrestricted use, distribution, and reproduction in any medium, provided the original author and source are credited

Protocol status: Working
We use this protocol and it's working

Created: June 16, 2025

Last Modified: June 23, 2025

Protocol Integer ID: 220581

Keywords: Non-Surgical Transcutaneous Intraglandular Injection, Cell and Cell-Free Therapies, Murine Submandibular Salivary Glands

Funders Acknowledgements:
Canadian Institute of Health Research
Grant ID: FBD-181455
Fonds de Recherche du Quebec
Grant ID: 324852

Abstract

Salivary Glands (SGs) are vital organs that are particularly prone to damage due to head and neck cancer radiation treatment, as well as other autoimmune disorders. SG hypofunction often impairs the production of saliva, which causes a significant loss of functioning for the oral cavity. There are a multitude of treatments for rescuing SG hypofunction; however, they are usually administered systemically, causing adverse effects and requiring higher doses of the agents. More localized approaches have demonstrated adequate potential as alternative treatments but are still in their initial stages of investigation. Here, we describe a step-by-step protocol for a localized delivery technique termed as the 'Intraglandular (IG) Non-Surgical Transcutaneous Injection' to deliver cell and cell-free therapies into the submandibular salivary glands (SMGs) of mice. This IG technique involves a direct injection into the SMGs without the need of invasive surgery and with the aid of imaging and anatomical landmarks.

Materials

Animals Used:

The Intraglandular Non-Surgical Transcutaneous injection was tested on C57Bl/6 and Non-Obese Diabetic (NOD) female mice, aged 20 weeks and with a bodyweight of 15-20 g. The mice are housed in clean conditions with ad libitum access to food and autoclaved distilled water.

Armamentarium (Fig 1):

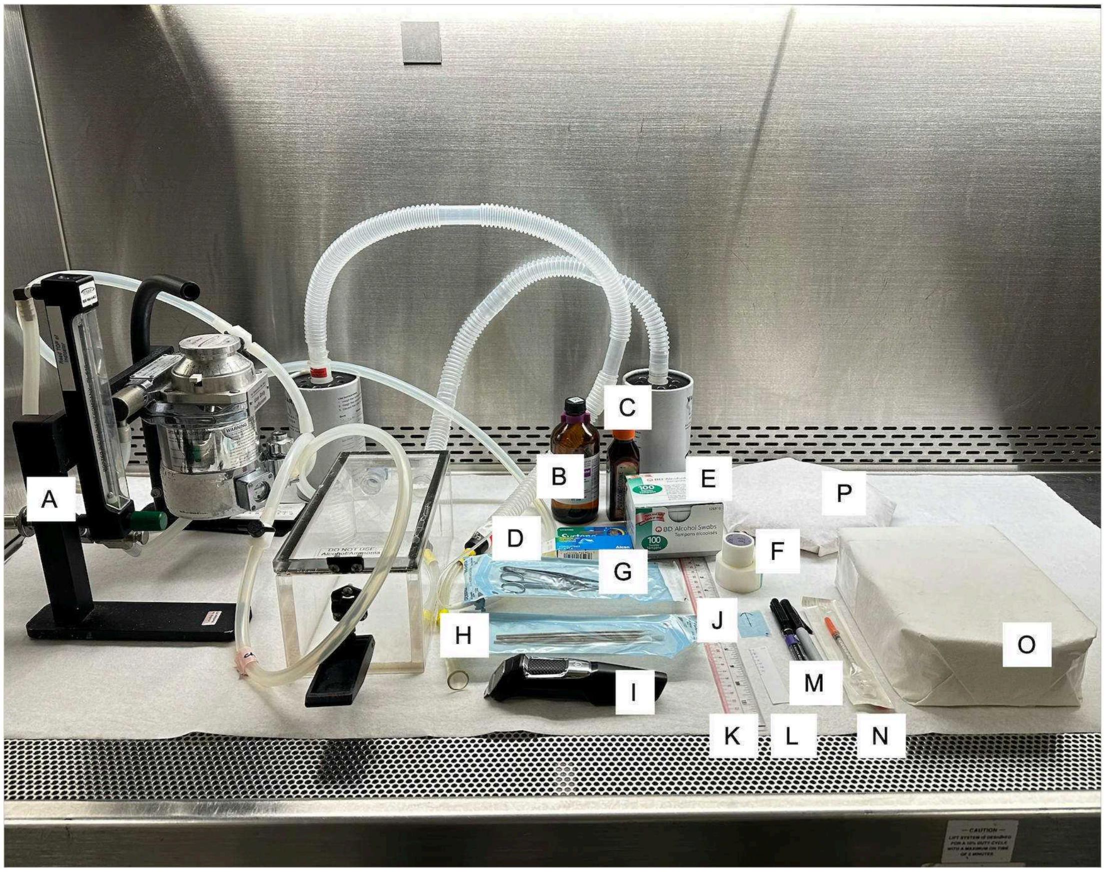

Figure 1: Armamentarium for the intraglandular injections: A. Inhalation anaesthetic unit (VetEquip Inc, USA); B. Isoflurane-USP; C. 10% Povidine-iodine solution; D. Lubricant ophthalmic ointment; E. 70% Ethanol swabs; F. Masking tapes; G. Surgical scissors, stainless steel; H. Anatomical forceps, fine, stainless steel; I. Electric shaving razor; J. Disinfected autoclavable plastic stencil; K. Disinfected ruler; L. Paper ruler; M. Fine tip markers; N. 29G, 12.7 mm, 0.5 ml, U-100 insulin syringe; O. Working platform; P. Heated recovery pad.

- All surgical procedures were performed under aseptic conditions. Surgical material was sterilized by autoclave and all surfaces were disinfected with 1:10 dilution of household bleach and 70% v/v ethanol.
- The operators donned personal protective equipment that included disposable laboratory coats, goggles, gloves, head caps, and masks.
- The required instruments and materials for the procedure were organized within the sterile field of the Biosafety Cabinet (BSC) hood.

- Female C57Bl/6 and NOD mice, aged 20 weeks, weighing 15-20g were given free access to water prior to the procedure.

Safety warnings

- All surgical procedures need to be performed under aseptic conditions, with autoclaved instruments and disinfected surfaces. Operators have to don personal protective equipment, including disposable laboratory coats, goggles, gloves, head caps, and masks.
- Procedures and organization of armamentarium to be performed within the Biosafety Cabinet.

Ethics statement

This protocol was conducted on mice, following all the guidelines laid by the Canadian Council on Animal Care and under protocols approved by the McGill University Animal Care Committee (Protocol #5330). Animal experiments conformed to the ARRIVE 2.0 guidelines. The procedure was performed under Isoflurane inhalation anaesthesia and all efforts were made to minimize suffering.

Before start

Note: Prior to Sedation, please refer to the "Materials" section for armamentarium set-up and preparation.

Sedation Procedure

1 Induce deep sedation to the mouse with an inductive dose of 4% (vol/vol) Isoflurane using an Isoflurane induction chamber (VetEquip Inc., USA).

2 Transfer the mouse onto an aseptic working platform in supine position and maintain sedation with a dose of 2% (vol/vol) Isoflurane using a nose cone. The depth of anesthesia can be evaluated by using the toe pinch reflex technique.

3 To avoid corneal drying, apply ophthalmic ointment on the upper and lower eyelids.

4 Restrain the mouse into position with masking tapes at a comfortable level of the nose cone exposing the lower lip and chin region (**Video-S1)**.

Anatomical Landmark Identification and SMG Localization

5 Swab the cervical area of the mouse (from the lower lip to approximately the sternum of the chest) with 70% Ethanol and shave the area using an electric shaver. Following this, clean the area with 10% Povidone-iodine solution (**Fig 2A, Video-S1**).

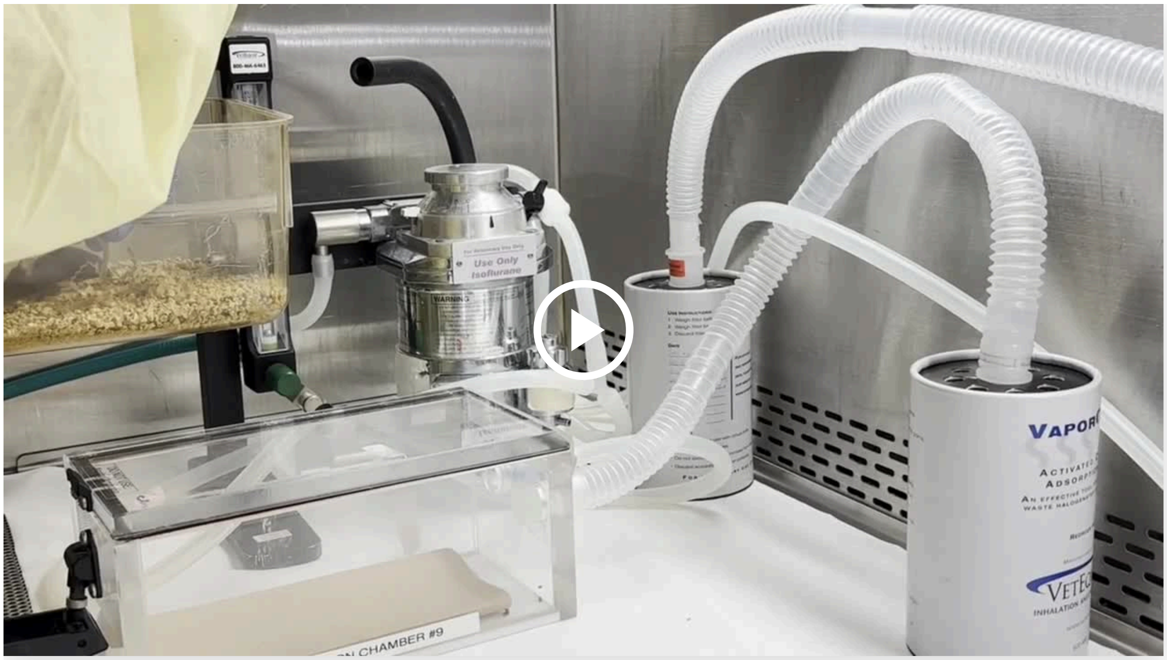

Video S1 : Procedure for Mouse Sedation and Preparation of Cervical Area

6 Plot the following landmarks using a fine-tipped marker (**Video-S2**)

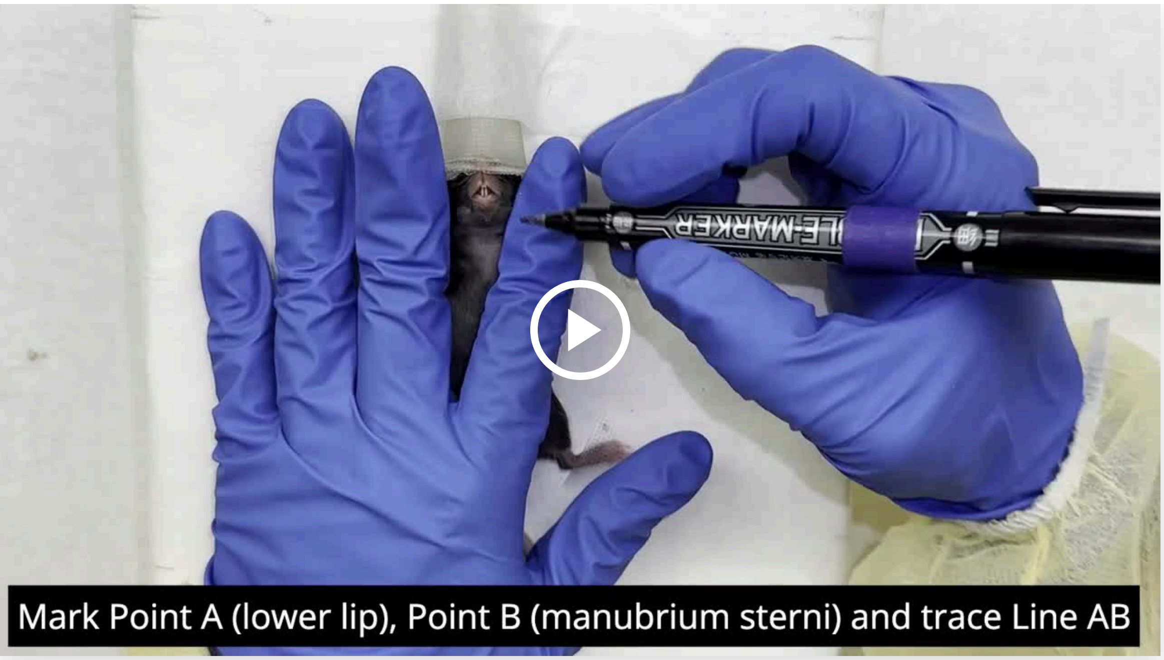

Video S2: Plotting of Anatomical Landmarks.

6.1 Draw a line from the midline of the lower lip (point A), passing through the inferior border of the mandible to the xiphoid process of the sternum (point B). This distance between Point A and Point B should be 19 mm, label this as 'Line AB,' (**Fig. 2B**).

6.2 Palpate and trace the lower border of the mandible from Point A to the angle of the mandible on either side. Measure 4 mm inferiorly from here bilaterally to reach the superior border of the SMGs.

6.3 From Point A, measure 9 mm inferiorly along the midline and mark Point C. Label this part of the midline as, 'Line AC'. From this point, measure 2 mm laterally on each side and mark it as Point D. This point represents the center of the SMGs on both sides and the site of injection. Label these bilateral lines as, 'Line CD.' (**Fig. 2C**)

6.4 For ease of reproducibility of these landmarks, an autoclavable plastic sheet can be used to develop a stencil. This is achieved by marking the above landmarks and their measurements using a fine-tipped black permanent marker. Use the stencil with the duplicated landmarks and measurements as a guide for future injections in multiple mice (**Fig. 2D**).

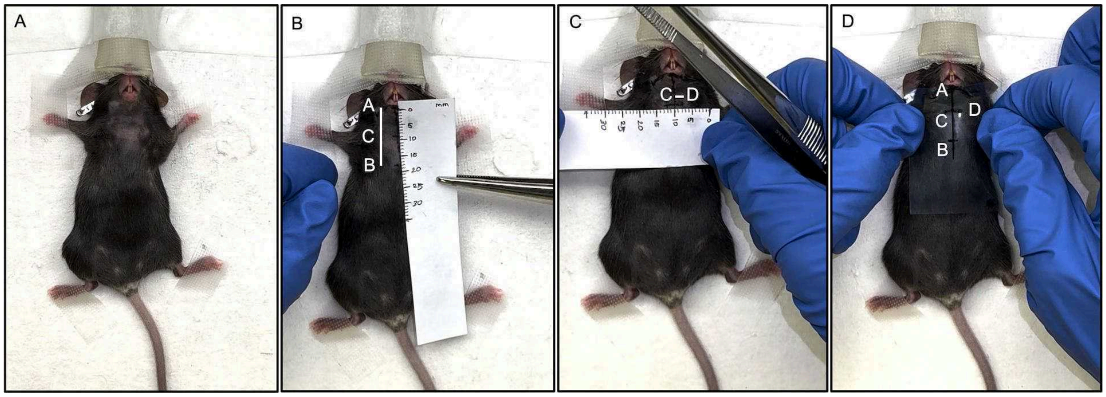

Figure 2 (A-D): Anatomical Landmark Identification. A. The cervical area of a sedated and restrained mouse was shaved and prepared for the procedure; B. The midline from the lower lip (point A) to Manubrium sterni (point B) (length of 19 mm). Point C was marked 9 mm inferiorly to point A; C. The sites of IG injections (points D) were measured 2 mm laterally on each side at point C; D. A plastic stencil was created as a guide for injections, using the above landmarks and measurements.

Intraglandular Injection Technique

7 Place the loaded syringe at one IG injection site (Point D), perpendicular to the mouse.

8 Insert the needle to a depth of 3 mm to reach the center of the SMG (**Fig 3A-B; Video – S3**). To aid with the insertion depth, 3 mm can be marked on the syringe needle.

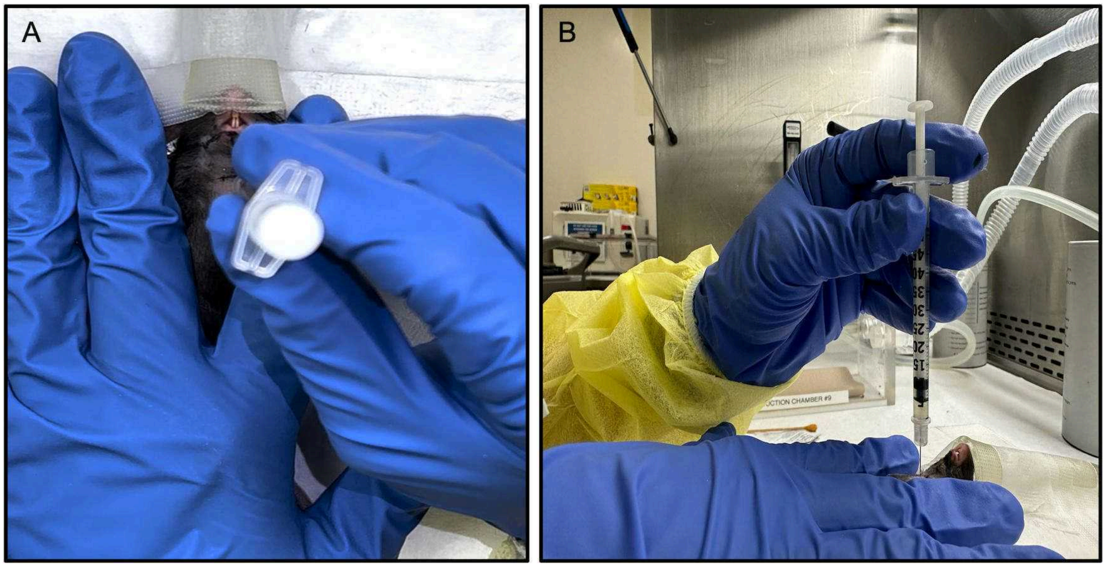

Figure 3 (A-B): The syringe loaded with the solution was placed at one IG injection site, perpendicular to the mouse (A. Bird's eye view; B. Lateral view).

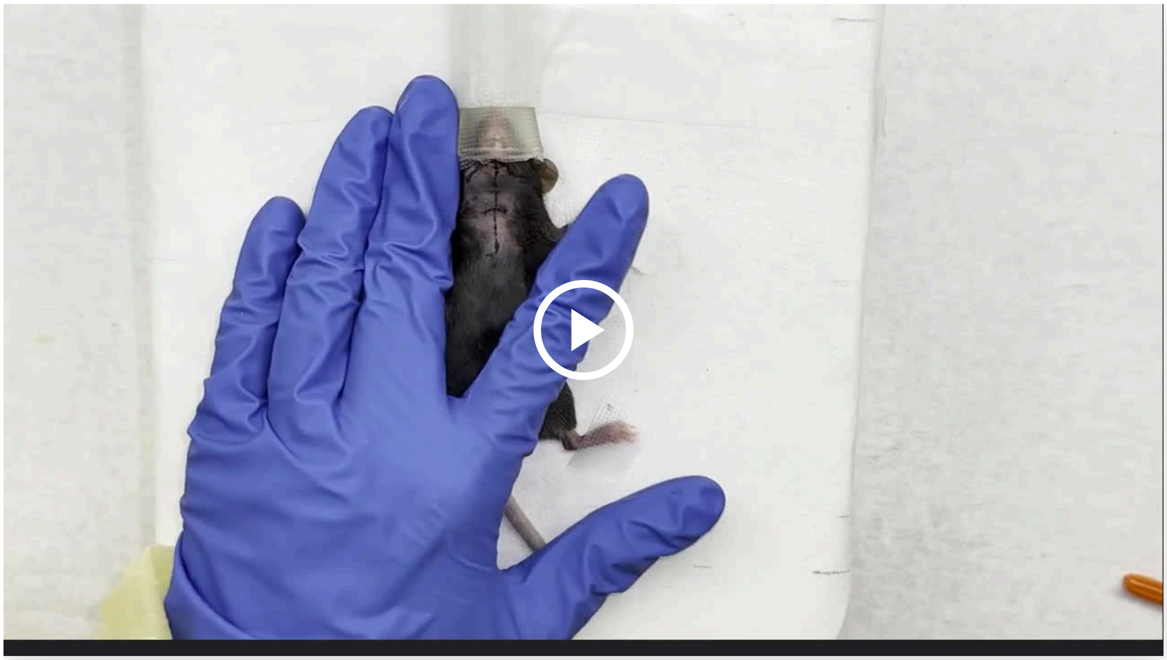

Video S3: Intraglandular Injection Procedure

9 Allow the needle to proceed past the recommended depth of 3 mm and then gently withdraw the syringe back upwards. While withdrawing, the solution can be gradually deposited to avoid gland perforation.

10 Gently massage the area of injection to dissipate the solution and repeat for the SMG on the other side.

Post-Operative Recovery

11 Turn off the Isoflurane and transfer the mouse to a hot pad for recovery. Monitor the mouse every hour for at least 5 hours and then monitor daily. Repeat the same steps mentioned above for the next mice.
